# MiRNA expression in the cartilage of patients with osteoarthritis

**DOI:** 10.1186/s13018-017-0542-y

**Published:** 2017-03-28

**Authors:** Marta Kopańska, Dariusz Szala, Joanna Czech, Natalia Gabło, Krzysztof Gargasz, Mateusz Trzeciak, Izabela Zawlik, Sławomir Snela

**Affiliations:** 1 Laboratory of Molecular Biology, Centre for Innovative Research in Medical and Natural Sciences, University of Rzeszow, Warzywna 1A, 35-959 Rzeszow, Poland; 2 Department of Genetics, Chair of Molecular Medicine, Faculty of Medicine, University of Rzeszow, Warzywna 1A, 35-959 Rzeszow, Poland; 3 Clinical Orthopedics and Traumatology Department in Clinical Hospital 2, 35-301 Rzeszow, Poland; 4 Data Analysis Laboratory, Centre for Innovative Research in Medical and Natural Sciences, Faculty of Medicine, University of Rzeszow, Rzeszow, Poland

**Keywords:** Osteoarthritis, Cartilage, miRNA, Expression, Pathogenesis, Patients

## Abstract

**Background:**

Osteoarthritis (OA), the most prevalent disease of articulating joints, is a complex multifactorial disease caused by genetic, mechanical, and environmental factors. In this research, we evaluated miRNA expression in OA.

**Methods:**

Forty tissue samples from 29 patients undergoing joint replacement for OA were evaluated. Tissue from two control patients undergoing hip replacement not related to OA was used as a control. Total RNA (containing miRNA species) from cartilage was isolated using a mirVana miRNA Isolation Kit. Expression of 19 miRNAs was assessed by real-time quantitative polymerase chain reaction.

**Results:**

Expression of four miRNAs, miR-138-5p, miR-146a-5p, miR-335-5p, and miR-9-5p, was significantly upregulated in OA tissues (patients vs. control group).

**Conclusions:**

These findings may contribute to disease prevention and the development of therapeutic targets for OA.

## Background

Osteoarthritis (OA) is a complex multifactorial disease caused by genetic, mechanical, and environmental factors, and it is the most prevalent disease of the articulating joints. Many environmental factors, such as hormones, diet, infections, injury, alcohol intake, and exposure to tobacco smoke, are associated with an increased risk of OA. Exposure of genetically susceptible individuals to such environmental factors may promote disease development [1]. Environmental factors induce epigenetic mechanisms that regulate genomic activity independently of changes in the DNA sequence and alter the expression of genes involved in disease development [2]. The three pillars of epigenetic regulation are DNA methylation, histone modification, and non-coding RNA species, including miRNA. The epigenetic mechanisms involved in the pathogenesis of OA are not understood [3]. Whether aberrant miRNA expression is associated with OA development is unclear. Many recent studies suggested that epigenetic events play a critical role in OA progression. Moreover, changes in gene expression have been observed in diseased cartilage [4]. Epigenetic mechanisms occur through miRNA expression. miRNAs comprise a large family of single-stranded, small, non-coding RNAs with a sequence length of 19 to 23 nucleotides. These molecules typically bind to the 3′ untranslated region of their target messenger RNAs (mRNAs) and repress protein expression by inhibiting mRNA translation and/or destabilizing mRNA. Micro RNAs are implicated in many cellular functions, such as apoptosis, lipid metabolism, malignant transformation, and differentiation [5].

Joint pain is the primary manifestation of OA. The complete role of miRNAs in OA is not known. It is the reason why we decided to extensively study this task. The prevalence of OA is increasing worldwide, prompting the search for reliable biomarkers of OA to aid in drug development. The design of novel therapeutic strategies, however, will require a better understanding of the function of differentially expressed miRNAs, as well as their target genes. In the present study, we evaluated miRNA expression in OA using real-time quantitative polymerase chain reaction (qPCR). The clinical treatment of OA is currently unsatisfactory. The results of this study provide a better understanding of the role of miRNA in OA and may contribute to disease prevention and the development of more precise therapeutic targets.

## Methods

Sample and clinical data collection was performed at the Clinical Orthopedics and Traumatology Department of Clinical Hospital no. 2 in Rzeszow, Poland. Samples were collected from femoral heads of 29 patients undergoing hip joint replacement planned surgeries of OA damaged tissue and compared to tissue obtained from femoral heads of two age-matched healthy patient controls undergoing surgery due to femoral neck fracture.

Cartilage was collected during hospitalization. Written informed consent to participate in the study was obtained from all participants. Critical points for sample collection were the proper diagnosis of OA and avoiding collection from sites with total cartilage loss. Clinical features of the participants were collected from the medical records based on interview about general information, previous injuries, BMI, occupational or sports activities, and clinical manifestations of OA; physical examination; and X-rays (Table 1). Samples were collected and prepared and then immediately stored in RNAse-free tubes in a –80 °C deep freezer until analysis. Ethics approval for this study was granted by the Bioethical Committee of the Medical Faculty of Rzeszow University (number: 5/01/2014).

**Table 1:** Clinical features of the participants

Characteristics	OA
Number of participants	29
Sex, male/female	16/13
Age, mean (range)	62.8 (23–83)
Body mass index (mean)	26.74
OA stage: early/late	10/19
Joints affected by disease (yes/no)	
Hip joint	29/0
Knee joint	8/21
Hands	4/25
Lumbar spine	10/19
Cervical spine	6/23
Feet	3/26
Surgery	8/21
Other diseases (yes/no)	
Heart	11/18
Circulatory system	18/11
Lung	2/27
Liver	1/28
Urinary system	4/25
Thyroid	3/26
Metabolic	5/25
Nervous system	2/27
Spine	13/26
Sciatica	8/21

### Total RNA isolation

For miRNA profiling, total RNA was purified and prepared. Total RNA (containing miRNA species) from cartilage was isolated using a mirVana miRNA Isolation Kit (Exiqon, Vedbaek, Denmark) following the manufacturer’s suggested protocol. For RNA isolated from frozen cartilage, the integrity and quantity of each sample were determined using a Bioanalyzer and NanoDrop™.

### Micro RNA real-time qPCR

Based on previously published reports and miRNA databases, we selected 22 miRNAs. In two healthy controls and 29 patients with OA, we analysed 22 miRNAs: let-7e-5p, miR-101-3p, miR-127-5p, miR-130a-3p, miR-138-5p, miR-146a-5p, miR-16-5p, miR-103a-3p, miR-423-5p, miR-191-5p, miR-193b-3p, miR-199a-3p, miR-210-3p, miR-21-5p, miR-222-3p, miR-22-3p, miR-27a-3p, miR-27b-3p, miR-335-5p, miR-454-3p, miR-9-5p, and miR-98-5p. The following three miRNAs, miR-103a-3p, miR-423-5p, and miR-191-5p, were used as endogenous normalization controls. All of these three miRNAs are recommended by Exiqon as good reference genes in cartilage samples and are found to be the stable normalizers.

Reverse transcription of 10 ng RNA was performed in 10-μl reactions using the miRCURY LNA™ Universal RT microRNA PCR, Polyadenylation and cDNA Synthesis Kit (Exiqon, Denmark). The complementary DNA (cDNA) was diluted 100 times and assayed in 10-μl PCR reactions according to the manufacturer’s protocol for miRCURY LNA™ Universal RT microRNA PCR; each microRNA was assayed once by qPCR on the microRNA Ready-to-Use PCR, Custom Pick and Mix Panel using ExiLENT SYBR® Green master mix. Negative controls in which the template was excluded from the reverse transcription reaction were processed in the same manner as the samples. Amplification was performed in a LightCycler® 480 RealTime PCR System (Roche) in 384-well plates. The amplification curves were analysed using Roche LC software, both for determination of the quantification cycle (Cq) by the 2nd derivative method and for melting curve analysis.

### Data analysis

**Fig. 1:**
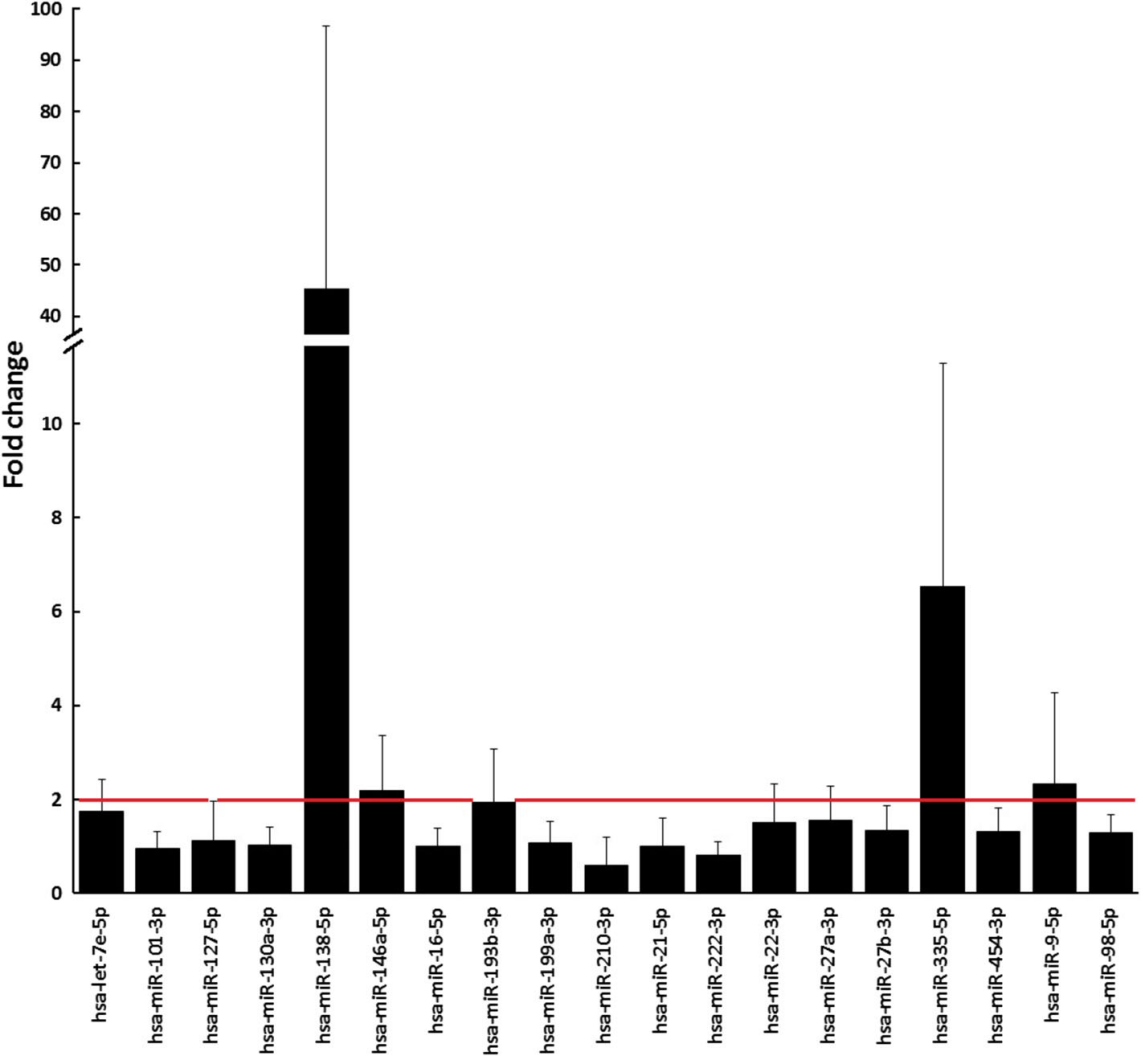
Cartilage miRNAs with differential expression in osteoarthritis. Mean fold change + standard deviation (SD). *Red line* cut-off value of fold change equals two

The amplification efficiency was calculated using algorithms implemented by LinReg software. All assays were inspected for distinct melting curves, and the melting temperature was confirmed to be within known specifications for the assay. Furthermore, the Cq of the assay had to be 3 Cqs less than that of the negative control, and less than 37 for the assay to be included in the data analysis. The Cq was calculated as the 2nd derivative. Using NormFinder, the best normalizer was found to be the average of assays detected in all samples (miR-103a-3p, miR-423-5p, and miR-191-5p). The following formula was used to calculate the normalized Cq values: normalized Cq = average Cq (*n* = 93) − assay Cq (sample). Relative expression of each gene was calculated by 2^−ΔΔCt^ formula [6]. The level of miRNA expression was classified as increased when the fold change is greater than two [7] (Fig. 1).

### Statistical analysis

Statistical analysis was performed using Statistica 12.5PL software (Statsoft, Poland) and PAST software (ver. 1.57). We compared microRNA expression between control and patient groups to create nonmetric multidimensional scaling, and then, an analysis of similarity was performed to evaluate hypothesized differences between groups of samples using a permutation/randomisation method of resemblance matrix plots for each of the datasets [8]. The distribution of variables was tested using the Shapiro-Wilk test and Kolmogorov-Smirnov test with Lilliefors correction. Because of the non-normal distribution of all analysed variables, we used a non-parametric test, the Mann-Whitney *U* test [9].

## Results

**Fig. 2:**
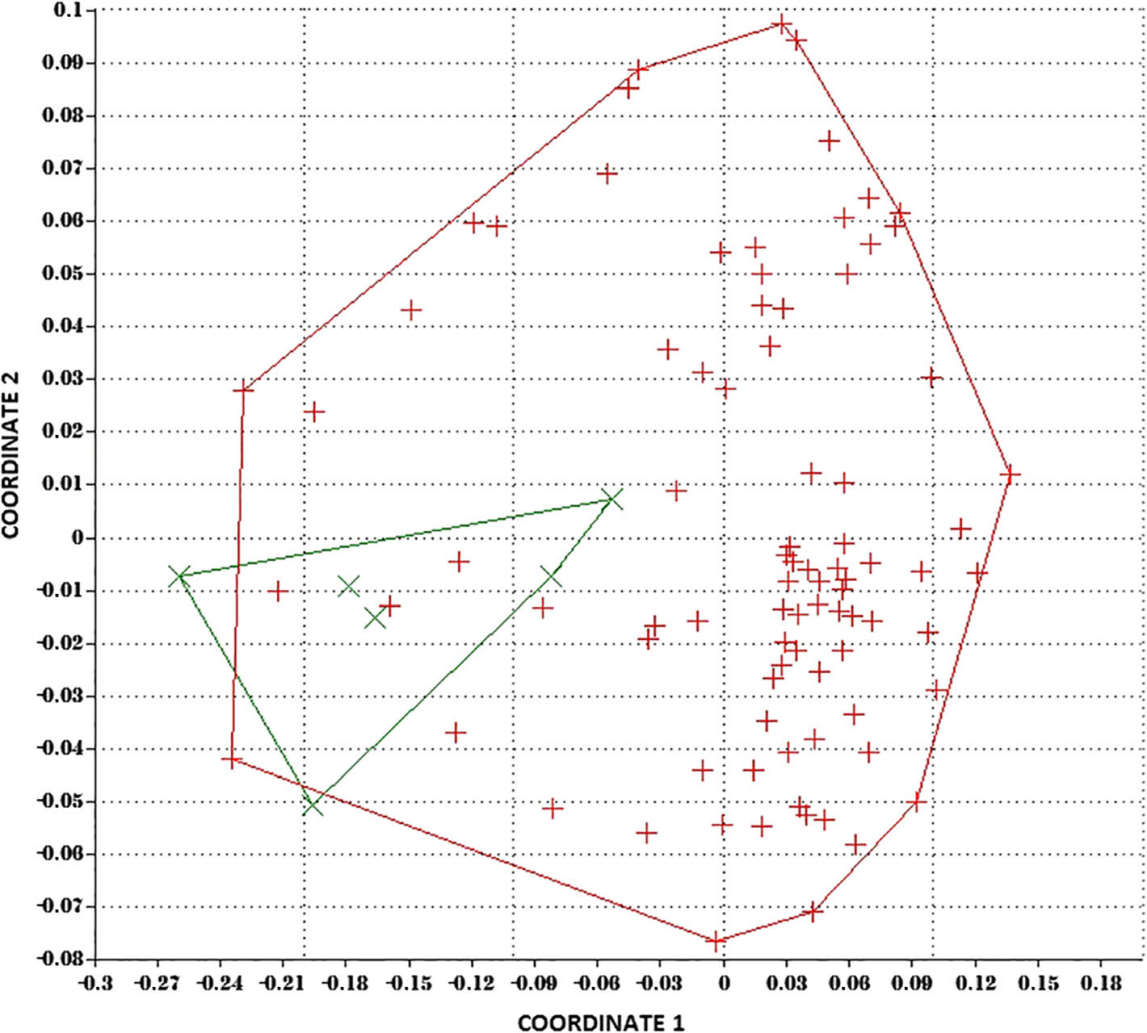
Multidimensional scaling plot demonstrating differences between patients (*red*) and controls (*green*) in expression of all miRNAs examined (analysis of similarity test *R* = 0.315; *p* = 0.002)

Multidimensional scaling and analysis of similarity revealed differences in the expression of all miRNAs between patients with OA and controls (Fig. 2). Analysis of similarity indicates a significantly different expression of mRNA between the two groups (*R* = 0.315; *p* = 0.002). Next, for all 19 miRNAs, the fold change was calculated. Of the 19 miRNAs, the expression of 4 (hsa-miR-138-5p, hsa-miR-146a-5p, hsa-miR-335-5p, and hsa-miR-9-5p) was higher than 2-fold change (OA patients compared with controls, Fig. 1). No statistically significant relationship was detected between the clinical data (Table 1) and the miRNA expression values.

## Discussion

**Fig. 3:**
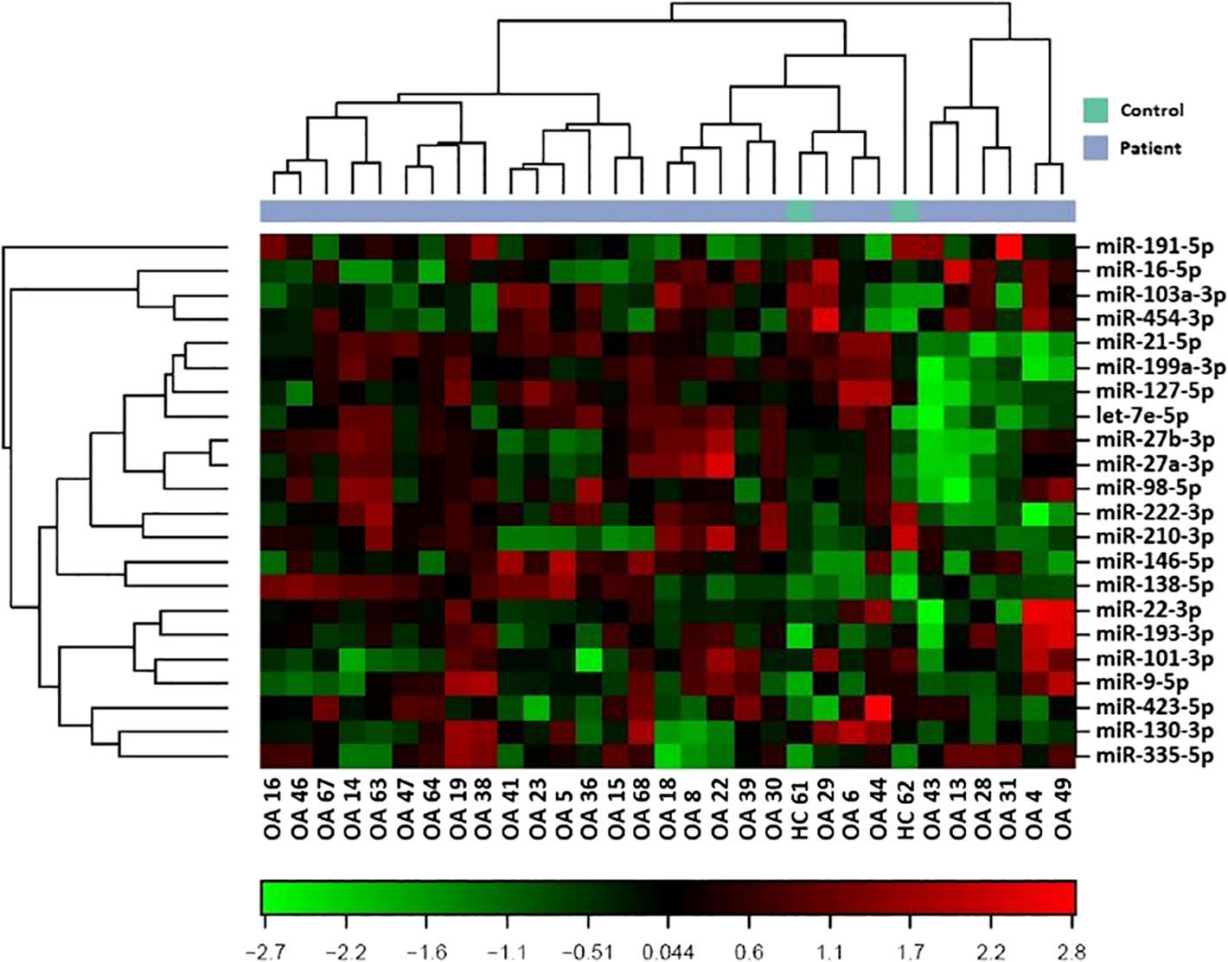
Unsupervised hierarchical clustering of differentially expressed miRNAs in the cartilage of the healthy controls and OA patients. Clustering was performed in all samples. Each row represents one miRNA, and each column represents one sample. The miRNA clustering tree is shown on the *left*. Normalized (dCq) values were used in the analysis. The colour scale illustrates the relative level of miRNA expression: *red* indicates expression level above the mean (315 times), *green* represents expression level below the mean (367 times)

Micro RNAs regulate diverse aspects of normal physiologic conditions. On the other hand, miRNA levels change in response to pathologic alterations, possibly contributing to the pathogenesis of degenerative diseases, such as OA [5]. Therefore, it is important to determine how miRNA expression is regulated in OA. Here, we found that the expression of four miRNAs (hsa-miR-138-5p, hsa-miR-146a-5p, hsa-miR-335-5p, and hsa-miR-9-5p) was upregulated in OA patients compared to that in controls (Fig. 3).

miR-146a is one of the first miRNAs reported to be differentially expressed in OA cartilage. mir-146a is a crucial determinant in knee joint homeostasis and pain symptoms associated with OA, maintaining the balance between the inflammatory response and expression of pain-associated factors in cartilage glial cells and synovium [10]. Yamasaki et al. [11] reported that miR-146a is highly expressed in cartilage with a low Mankin grade, and its expression is decreased in proportion to the level of matrix metalloproteinase-13 expression. miR-146a is also expressed in all chondrocyte layers, especially in the superficial layers, while miR-146a-expressing cells are lightly distributed in the deep zone, where the matrix appears normal. Interleukin-1 beta induces the expression of miR-146a when the first degenerative changes appear and may play a key role in the inhibition of catabolic factors through negative feedback, including downregulation of the adaptor proteins IRAK1 and TRAF6, in early-stage OA cartilage. In late-stage OA, expression of miR-146a is low, which leads to progressive degradation of the cartilage due to decreased suppression of catabolic signals [9].

In our study, expression of miR-138-5p was upregulated which is consistent with the results of Yuan et al. They suggested that miR-138-5p in interleukin-1 beta (IL-1β) induced extracellular matrix (ECM) degradation of OA cartilage. The expression levels of miR-138-5p are significantly increased in OA cartilage and chondrocytes, as well as in response to IL-1β stimulation. The results indicate that miR-138-5p might be involved in the pathogenesis of OA. miR-138-5p directly modulates the expression of FOXC1, contributing to IL-1β-induced ECM degradation in chondrocytes [12].

Patients with OA exhibit early signs of cartilage degradation, synovial inflammation, and altered bone structure even before the disease manifests clinically [13]. Circulating miRNAs are easily accessible and stable [14, 15], making them convenient for daily clinical monitoring. A study of a large cohort representative of the general community by Beyer et al. [16] provided evidence that specific miRNAs, such as let-7e, can serve as valuable biomarkers for OA.

Expression of miR-146a is also reported in OA chondrocytes; mechanistically, miR-146a is apparently responsive to IL-1b signalling and may be involved in the pain-related pathophysiology of OA [10, 11, 17]. This miRNA was detected in our screening, but was not differentially expressed between cartilage regions. The main difference between miR-146a and miR-146b is the presence of two nucleotides located on different chromosomes. Thus, it is possible that the miRNA homologues mentioned above are differentially regulated during chondrogenesis and have distinct functions associated with the control of developmental processes and maintenance of homeostasis in mature tissue.

In the present study, we reviled increased expression of miR-9. According to Gu et al. results, targeting of miR-9 to NF-κβ1 may enhance proliferation and suppress apoptosis of knee OA chondrocytes through modification of IL-6 and MMP-13. Moreover, miR-9 and NF-κβ1 could potentially serve as diagnostic biomarkers and therapeutic targets for patients with knee OA [18]. Song et al. in their study found that protogenin (PRTG) is regulated by miR-9, resulting in an inhibition of cell proliferation and survival in chondrogenic progenitors and articular chondrocytes. Reduction of miR-9 induction, which results in increased PRTG levels in OA pathogenesis, may be responsible for chondrocyte apoptosis, a typical hallmark of OA [19].

We detected significant changes in the differential expression patterns of miR-335-5p and the less abundant 3p strand (miR-335*). We have observed a sharp decline in miR-335-5p expression during transforming growth factor β3-induced chondrocyte differentiation of hMSCs (unpublished data), suggesting a potential role for the 5p strand of miR-335 in regulating genes to maintain a more progenitor-like phenotype. Supporting this view, Tome et al. [20] reported that miR-335-5p downregulation is required for MSC differentiation toward the osteogenic or adipogenic lineage; overexpression of this miRNA inhibits MSC differentiation. On the other hand, Zhang et al. [21] reported opposite effects of miR-335-5p in regulating osteogenesis, but this may be explained by the fact that they used cell lines as opposed to primary MSCs. The present study is the first to report the presence of miR-138 in the cartilage and that higher expression of this miRNA is associated with differentiated and hypertrophic chondrocytes compared with precursor cells. These expression patterns suggest a potential role of miR-138 in regulating specific phases of chondrocyte differentiation. Other studies indicate that overexpression of miR-138 inhibits osteogenic and adipogenic differentiation [22, 23]. Interestingly, it has also been demonstrated that miR-138 promotes induced pluripotent stem cell generation via regulation of p53 [24], indicating that miR-138 can control cellular differentiation and may function through different mechanisms depending on the tissue microenvironment. How this miRNA regulates chondrogenesis and the mechanisms involved are important questions for future studies.

## Conclusions

Together, the findings of the present study indicate a correlation between the expression of four miRNAs (hsa-miR-138-5p, hsa-miR-146a-5p, hsa-miR-335-5p, and hsa-miR-9-5p) and OA. Changes in the expression of these four miRNAs were not significantly related to the clinical data (Table 1) however, or the expression of selected miRNAs. Therefore, further studies are needed to assess the usefulness of monitoring cartilage miRNAs for miRNA*-*based prognostic and therapeutic approaches.

## Abbreviations

Cq: Quantification cycle
IGFBP-5: Insulin-like growth factor-binding protein 5
IL-1β: Interleukin-1 beta
IRAK1: Interleukin-1 receptor-associated kinase 1
MMP-13: Matrix metalloproteinase 13
OA: Osteoarthritis
TRAF6: TNF receptor-associated factor 6
