# Erratum to: MiRNA expression in the cartilage of patients with osteoarthritis

**DOI:** 10.1186/s13018-017-0577-0

**Published:** 2017-06-13

**Authors:** Marta Kopańska, Dariusz Szala, Joanna Czech, Natalia Gabło, Krzysztof Gargasz, Mateusz Trzeciak, Izabela Zawlik, Sławomir Snela

**Affiliations:** 1 Laboratory of Molecular Biology, Centre for Innovative Research in Medical and Natural Sciences, University of Rzeszow, Warzywna 1A, 35-959 Rzeszow, Poland; 2 Department of Genetics, Chair of Molecular Medicine, Faculty of Medicine, University of Rzeszow, Warzywna 1A, 35-959 Rzeszow, Poland; 3 Clinical Orthopedics and Traumatology Department in Clinical Hospital 2, 35-301 Rzeszow, Poland; 4 Data Analysis Laboratory, Centre for Innovative Research in Medical and Natural Sciences, Faculty of Medicine, University of Rzeszow, Rzeszow, Poland

## Erratum

In the original publication of this article [1], was an error in the abstract and the methods section which was published with an incorrect kit citation.

The error 1:

Total RNA (containing miRNA species) from cartilage was isolated using a mirVana miRNA Isolation Kit.

Should be read instead:

Total RNA (containing miRNA species) from cartilage was isolated using a miRCURYTM Isolation Kit.

The error 2:

For miRNA profiling, total RNA was purified and prepared. Total RNA (containing miRNA species) from cartilage was isolated using a mirVana miRNA Isolation Kit (Exiqon, Vedbaek, Denmark) following the manufacturer’s suggested protocol.

Should be read instead:

For miRNA profiling, total RNA was purified and prepared. Total RNA (containing miRNA species) from cartilage was isolated using a miRCURYTM Isolation Kit (Exiqon, Vedbaek, Denmark) following the manufacturer’s suggested protocol.

## References

[1] MiRNA expression in the cartilage of patients with osteoarthriti. J Orthop Surg Res. 2017;12:51. doi: 10.1186/s13018-017-0542-y 10.1186/s13018-017-0542-y PMC5371266 28351380

